# Social status and cardiovascular disease: a Mediterranean case. Results from the Italian Progetto CUORE cohort study

**DOI:** 10.1186/1471-2458-10-574

**Published:** 2010-09-24

**Authors:** Carla Fornari, Chiara Donfrancesco, Michele A Riva, Luigi Palmieri, Salvatore Panico, Diego Vanuzzo, Marco M Ferrario, Lorenza Pilotto, Simona Giampaoli, Giancarlo Cesana

**Affiliations:** 1 Research Centre on Public Health, Department of Clinical and Preventive Medicine, University of Milano-Bicocca, Monza, Italy; 2 Laboratory of Epidemiology and Biostatistics, National Institute of Public Health, Rome, Italy; 3 Department of Clinical and Experimental Medicine, University Federico II of Naples, Naples, Italy; 4 Centro per la Prevenzione Cardiovascolare, Azienda Socio-Sanitaria 4 Medio Friuli and Agenzia Regionale della Sanità, Udine, Italy; 5 Department of Clinical and Biological Science, University of Insubria, Varese, Italy

## Abstract

**Background:**

Social factors could offer useful information for planning prevention strategy for cardiovascular diseases. This analysis aims to explore the relationship between education, marital status and major cardiovascular risk factors and to evaluate the role of social status indicators in predicting cardiovascular events and deaths in several Italian cohorts.

**Methods:**

The population is representative of Italy, where the incidence of the disease is low. Data from the Progetto CUORE, a prospective study of cohorts enrolled between 1983-1997, were used; 7520 men and 13127 women aged 35-69 years free of previous cardiovascular events and followed for an average of 11 years. Educational level and marital status were used as the main indicators of social status.

**Results:**

About 70% of the studied population had a low or medium level of education (less than high school) and more than 80% was married or cohabitating. There was an inverse relationship between educational level and major cardiovascular risk factors in both genders. Significantly higher major cardiovascular risk factors were detected in married or cohabitating women, with the exception of smoking. Cardiovascular risk score was lower in married or cohabitating men. No relationship between incidence of cardiac events and the two social status indicators was observed. Cardiovascular case-fatality was significantly higher in men who were not married and not cohabitating (HR 3.20, 95%CI: 2.21-4.64). The higher cardiovascular risk observed in those with a low level of education deserves careful attention even if during the follow-up it did not seem to determine an increase of cardiac events.

**Conclusions:**

Preventive interventions on cardiovascular risk should be addressed mostly to people with less education. Cardiovascular risk score and case-fatality resulted higher in men living alone while cardiovascular factors were higher in women married or cohabitating. Such gender differences seem peculiar of our population and require further research on unexpected cultural and behavioural influences.

## Background

In several studies social status, defined through various variables - education, occupation, income, etc - taken alone or together, were inversely associated with cardiovascular disease (CVD )[1,2]. In Italy this relationship has been reported in the North [3,4] and in the Centre of the country [5,6], but not in the South.

Among the most important indicators of social status are education and marital status. Higher educational levels resulted protective against all causes of mortality [1] and circulatory events [7]. Various studies reported an inverse gradient in incidence and mortality for coronary and stroke events by educational level [8-11]. The lower cardiovascular risk in people with a higher level of education is usually explained as a result of the role that education plays in understanding and achieving healthy lifestyles.

Marital status has been found to be associated with general mortality in many studies conducted in the United States[12], European countries [13,14] and Japan [15]. In particular, elevated mortality risk was associated with single status for both genders, whereas the link between mortality and divorce or widowhood was found only in men [14,15]. Indeed, unmarried men and women tended to engage in more unhealthy behaviours (smoking, physical inactivity and low interest in health screening) and to experience more psychological distress [16]. On the contrary, the social ties and family support, provided by marriage, seemed to reduce the risk of mortality from major disease of all causes [12].

From the second half of the 1970 s, in Italy as in most Western European countries, CVD mortality decreased across the whole country, mainly in the Northern regions, where rates were originally higher [17,18]. During the Nineties almost two thirds of the decrease in coronary mortality were due to the reduction in 28-day case-fatality [19]. The incidence of coronary disease decreased too, but less than mortality. Case-fatality decreased both for total events (largely explained by the reduction of out-of-hospital deaths) and for hospitalised cases (in the same period, medical management of cardiovascular events underwent an impressive improvement) [20]. Only a few studies documented the incidence and case-fatality of cerebrovascular disease in Italy [21,22]. Rates seemed to decline but comparison in time was difficult due to the lack of standard criteria of classification and the discontinuity of data collection. Major cardiovascular risk factors (total cholesterol, blood pressure and cigarette smoking) showed a progressive decrease, even in the absence of systematic and active preventive interventions [23].

The aims of this study were to explore the relationship between education, marital status and major cardiovascular risk factors and to evaluate the role of such indices of social status in predicting circulatory events and deaths in Italian cohorts. The studied population is characterized by a relatively low incidence of the disease, usually attributed to the Mediterranean diet [24-26] and, so far, differently from Northern Europe, not clearly or weakly influenced by social conditions.

## Methods

### Population

Twelve population-based cohorts from the North and the Centre-South of Italy involved in the Progetto CUORE were investigated [27]. Within the frame of the WHO MONICA Project, six samples were selected from 25-64 year-old residents of two Northern Italian areas: in Brianza (Lombardy) the cohorts were enrolled in 1986, 1990, 1993 and in Friuli in 1986, 1989, 1994 [28]. The PAMELA Study was a random sample of 25-74 year-old residents of the town of Monza (Lombardy) investigated in 1990-93 by the MONICA Brianza team [29]. The Friuli Emostatico study was a random sample of the 45-64 year-old residents of the town of Udine in Friuli, enrolled in 1995-96 and investigated by the MONICA Friuli team. The MATISS study selected three independent cohorts from the general population in the age group of 20-69 years of the Latina Province, an area located in the south of Rome, in 1984, 1987 and 1993 respectively [30]. Finally, the Progetto ATENA cohort was selected from the 30-69 year-old female population of Naples in 1993-1997 [31]. These cohorts were originally included in the Progetto CUORE to assess cardiovascular risk factor prevalence and CVD morbidity and mortality time trends from the first half of the 1980s to the first half of the 1990s. The present analysis is restricted to 13127 women and 7520 men in the age group of 35-69 years and free of previous cardiovascular events at baseline [27]. The follow-up of major cardiovascular events continued until December 2002, for an average period of 11 years: the percentage of losses to follow-up is less than 5% in all cohorts. The CUORE Project received approval from the Ethics Committee of the Istituto Superiore di Sanità in 2006, 2008 and 2009. At the baseline visit all the participants gave the consent for the follow-up of mortality and cardiovascular morbidity in the respect of the Italian privacy low.

### Cardiovascular risk factors and social status

Cardiovascular risk factors were measured at baseline adhering to the procedures and quality standards of the WHO-MONICA project [28]. Briefly, blood pressure was measured twice on the right arm with a standard mercury sphygmomanometer on sitting subjects, at rest for at least 10 minutes. Systolic and diastolic blood pressures were recorded as the first and fifth phase of Korotkoff sounds. The average of the two measurements, taken five minutes apart, was used as the study variable. Venous blood specimens were taken after overnight fasting. Serum total and HDL-cholesterol were assayed by the enzymatic method in the collaborating laboratories. Triglycerides were determined using the GPO_PAP enzymatic colorimetric method or the Trinder method.

Weight and height were measured in participants without shoes and with light clothing and they were used to compute the body mass index (BMI). A standard questionnaire was administered to collect information on cigarette smoking, diabetes, medications and history of CVD. Smoking status was classified as a dummy variable which identify smokers (current smokers or subjects who stopped smoking less than 12 months ago) and non-smokers (never smoked or past smokers who stopped smoking at least 12 months ago at baseline). Diabetes mellitus was defined using self-reported diagnosis, information on insulin and oral hypoglycaemic treatments and fasting blood glucose (FBG) exceeding 126 mg/dl. Individual 10-year global cardiovascular risk was identified using Progetto CUORE predictive equation of coronary events [27]. This equation for prediction of cardiovascular events was calibrated on the 35-69 year-old Italian population and included the following risk factors: age, total and HDL cholesterol, systolic blood pressure, cigarette smoking, diabetes mellitus and drug treatment for hypertension.

Educational level and marital status were collected by a standard questionnaire at baseline and they were used as the indicators of social status. The choice of education was due to its simplicity as an index, importance in determining socioeconomic conditions, and, finally, stability over time. Educational level was categorized into three groups, taking into account the effect of changes in the education system over time. A study on the definition of the school system for the Brianza MONICA cohorts remarked that the schooling cut-off points stabilized to 8.5 and 12.5 years only for men born after 1952 and women born after 1960 [32]. Otherwise cut-off points were 5 and 8 years. As men born after 1952 or women born after 1960 were only the 3% of the studied population, educational levels were defined as follows: "High" included high school, college or university (> 8 years), "Medium" secondary school (5-8 years) and "Low" primary school (< 5 years).

Marital Status was categorised into four classes: subjects married or cohabitating, single, separated or divorced and widowed. Due to the low number of single, separated, divorced and widowed subjects in the studied population ( < 5%) and according to previous reported association between marital status and CVD risk [33] we grouped marital status into two classes: subjects "married or cohabitating" and subjects with "other living arrangements" as single, separated or divorced and widowed. We ascertained that these people lived alone.

### Ascertainment of cardiovascular events

Fatal and non-fatal major cardiovascular events were collected until December 2002, except for the ATENA cohort which concluded the follow-up in December 2001. Deaths were identified through an active collection of information on subject vital status from municipalities and death certificates from Local Health Districts. Causes of death with International Classification of Disease (ICD-IX) codes 410-414, identified a suspected coronary event and ICD-IX codes 430-432, 434-436, 437 a suspected stroke event. Non-fatal coronary and stroke events were identified in the regional hospital discharge datasets, through deterministic or, when necessary (incomplete data), probabilistic record linkage between the name of the enrolled subjects and the diagnosis coded ICD-IX 410-411, 430-432, 434-436 as discharge diagnosis or ICD-IX 36.0-9, 38.12 as procedure codes. Re-screening of cohorts, phone and postal interviews or MONICA registers were also used to identify suspected events in the cohort enrolled before the 1990s, when hospital discharge registration was inadequate. Suspected events were validated according to the MONICA diagnostic criteria [28]. Case-fatality for incident events was defined by death within 28 days from the onset of acute symptoms.

Events validated as definite and possible myocardial infarctions (MIs), coronary revascularization, silent MIs and sudden cardiac deaths were included in the analysis as coronary end-points; definite stroke and carotid arterectomy as stroke end-points.

### Statistical analysis

The relationship between cardiovascular risk factors, education and marital status was studied at baseline stratifying by gender. Mean values and proportions were age-adjusted using direct standardization method with the 1995 European population, age group 35-69 years. ANOVA for continuous variables and Chi-square test were used to assess statistical significance. Bonferroni correction for multiple comparisons was used.

Hazard ratios (HR) and 95% confidence intervals for cardiovascular events or case-fatality in social categories were computed using Cox proportional hazard models adjusted for age, cohort and cardiovascular risk factors (systolic blood pressure, total cholesterol, HDL cholesterol, diabetes, smoking habits, antihypertensive medication). Cox models for marital status were adjusted also for educational level. Reference groups for HR calculation were "High education" for education classes and "Married or cohabitating" for marital status. The analysis was performed also stratifying by geographical area: North and Centre-South of Italy. No differences were found where not specified. Statistical analysis was carried out using the SAS software and 0.05 as significance level.

## Results

About 70% of the population studied had a low or medium level of education and more than 80% was married or cohabitating, both for men and women (Table 1).

**Table 1:** Baseline characteristics, men and women 35-69 years-old free of previous CVD.

	Men (N = 7520)		Women (N = 13127)	
	n	%	n	%
*Age groups*				
*35-39*	1095	14.6	1524	11.6
*40-49*	2437	32.4	4823	36.7
*50-59*	2403	32.0	4552	34.7
*60-69*	1585	21.1	2228	17.0
Missing	0	0.0	0	0.0
				
*Educational level*				
High	2262	30.1	3788	28.9
Medium	2091	27.8	3557	27.1
Low	3017	40.1	5439	41.4
Missing	150	2.0	343	2.6
				
*Marital Status*				
Married or cohabitating	6643	88.3	10767	82.0
Other living arrangements	814	10.8	2276	17.3
Missing	63	0.8	84	0.6

In Tables 2 and 3 main cardiovascular risk factors are described by educational level and marital status, respectively in men and women. There was a significant relationship between educational level and several major cardiovascular risk factors. Men in the low education category had significantly lower levels of total cholesterol and prevalence of treatment for hypertension than men in the high and medium education categories but higher levels of triglycerides and prevalence of diabetes. BMI and proportion of smokers increased from the high education group to the low. Highly educated men had lower levels of diastolic blood pressure than the other men and lower systolic blood pressure than less educated men. In women, blood pressure, triglycerides, BMI levels and prevalence of diabetes and antihypertensive treatment had an increasing trend from high to low levels of education. Total, HDL cholesterol and the proportion of smokers decreased with a lower educational level. Individual global cardiovascular risk was lower in highly educated women. Highly educated men had higher global risk than less educated men.

**Table 2:** Cardiovascular risk factors in relation to social status indicators.

MEN	EDUCATIONAL LEVEL			MARITAL STATUS	
	High	Medium	Low	Married or Cohabitating	Other living arrangements
	*mean (s.d.)*	*mean (s.d.)*	*mean (s.d.)*	*mean (s.d.)*	*mean (s.d.)*
Systolic blood pressure (mmHg)*	137.5 (17.3)^•^	139.3 (18.8)	138.0 (19.3)	138.6 (18.5)	139.9 (19.5)
Diastolic blood pressure (mmHg)*	86.0 (10.3)^•^°	87.0 (11.1)	87.2 (11.2)	86.8 (10.8)	86.8 (11.8)
Total Cholesterol (mg/dl)*	226.2 (43.0)°	226.4 (46.6)'	222.2 (42.5)	224.8 ( 44.0)	224.0 (45.1)
HDL Cholesterol (mg/dl)	49.8 (12.9)	50.6 (14.4)	49.7 (13.7)	50.2 (13.7)	50.8 (14.2)
Triglycerides (mg/dl)*	141.9 (93.4)°	149.5 (115.2)'	160.8 (112.8)	151.7 (106.6)	156.7 (121.9)
BMI (kg/m^2^)*^^^	26.3 (3.4)^•^°	26.6 (3.6)'	27.0 (3.7)	26.8 (3.6)	26.3 (3.7)
Cardiovascular risk score^§*^ ^	7.4 (3.6)°	7.7 (4.6)	7.9 (6.3)	7.7 (5.0)	8.2 (5.7)
	*n (%)*	*n (%)*	*n (%)*	*n (%)*	*n (%)*
Diabetes (Yes/No)*^^^	85 (4.6)°	106 (5.2)'	233 (6.8)	370 (5.5)	58 (7.6)
Smoke (Yes/No)*	760 (31.0)^•^°	815 (39.3)'	1371 (47.5)	2639 (39.7)	344 (41.7)
Antihypertensive Medication (Yes/No)*	164 (11.0)°	193 (10.8)'	334 (8.7)	622 (9.5)	77 (10.1)

Men 35-69 years-old, free of previous CVD.

Age-adjusted means and percentages using European Population 1995. s.d.=standard deviation.

*p < 0.05. ANOVA for continuous variables (test F) and Chi-squared test for categorical variables among educational levels. Bonferroni correction for multiple comparisons was used: ^• ^High vs Medium; ° High vs Low; ' Medium vs Low.

^^ ^p < 0.05. ANOVA for continuous variables (test F) and Chi-squared Test for categorical variables among marital status categories.

^§ ^Cardiovascular risk score calculated using Progetto CUORE Study equation.

**Table 3:** Cardiovascular risk factors in relation to social status indicators.

WOMEN	EDUCATIONAL LEVEL			MARITAL STATUS	
	High	Medium	Low	Married or Cohabitating	Other living arrangements
	*mean (s.d.)*	*mean (s.d.)*	*mean (s.d.)*	*mean (s.d.)*	*mean (s.d.)*
Systolic blood pressure (mmHg)*^^^	132.3 (17.0)^•^°	135.5 (18.7)'	138.2 (20.4)	136.3 (19.0)	134.3 (19.3)
Diastolic blood pressure (mmHg)*^^^	80.6 (9.5)^•^°	82.4 (10.2)'	85.2 (11.3)	83.4 (10.7)	82.2 (10.4)
Total Cholesterol (mg/dl)*	233.3 (41.5)^•^°	230.5 (41.3)'	222.4 (41.2)	227.0 (41.3)	227.7 (43.0)
HDL Cholesterol (mg/dl)*^^^	63.5 (15.7)^•^°	60.8 (15.6)'	56.7 (14.2)	59.5 (15.4)	60.4 (15.1)
Triglycerides (mg/dl)*^^^	106.1 (55.2)^•^°	110.3 (64.4)'	121.6 (70.4)	116.1 (65.1)	110.6 (65.0)
BMI (kg/m2)*^^^	25.6 (4.0)^•^°	26.7 (4.4)'	28.4 (4.9)	27.3 (4.7)	26.5 (4.7)
Cardiovascular risk score^§* ^	*2.6 (1.9)*^•^°	*2.9 (2.2)*	*2.8 (2.8)*	*2.8 (2.2)*	*2.8 (3.2)*
	*n (%)*	*n (%)*	*n (%)*	*n (%)*	*n (%)*
Diabetes (Yes/No)*	64 (2.2)^•^°	100 (3.4)'	312 (5.2)	392 (4.2)	94 (3.5)
Smoke (Yes/No)*^^^	1371 (34.0)^•^°	1096 (30.0)'	855 (16.4)	2730 (23.9)	647 (31.7)
Antihypertensive Medication (Yes/No)*	333 (11.3)^•^°	448 (14.8)'	1054 (17.3)	1481 (15.4)	411 (14.2)

Women 35-69 years-old, free of previous CVD.

Age-adjusted means and percentages using European Population 1995. s.d.=standard deviation.

*p < 0.05. ANOVA for continuous variables (test F) and Chi-squared test for categorical variables among educational levels. Bonferroni correction for multiple comparisons was used: ^• ^High vs Medium; ° High vs Low; ' Medium vs Low.

^^ ^p < 0.05. ANOVA for continuous variables (test F) and Chi-squared Test for categorical variables among marital status categories.

^§ ^Cardiovascular risk score calculated using Progetto CUORE Study equation.

The relation between marital status and cardiovascular risk factors was more moderate. Marriage or cohabitation were protective conditions for diabetes in men, but not in women. Major cardiovascular risk factors, such as high blood pressure, low HDL cholesterol, triglycerides and BMI, were higher in married or cohabitating women than in women with other living arrangements. The opposite was true for smoking (23.9% vs 31.7%). BMI was higher also in married or cohabitating men. No differences in the cardiovascular risk score were observed by marital status in women, while men living alone showed higher risk than men who were married or cohabitating.

During the follow-up, 718 cardiovascular incident events for men and 364 for women were detected and respectively 217 and 120 were fatal events. No relationship between events and social status indicators, educational level and marital status, was observed in either gender (Table 4), but cardiovascular case-fatality was significantly higher in men living alone (HR 3.20, 95%CI: 2.21-4.64) (Table 5). This relationship was detected also for stroke and coronary case-fatality considered separately (data not shown). Stratifying the study population by geographical area (Tables 4 and 5), in the Northern regions of Italy the incidence of cardiovascular events was higher in less educated men than in highly educated men (HR 1.41, IC 95% 1.10-1.81). This relationship was not found in the other regions. Case-fatality was higher for men living alone both in the North and in the Centre-South of Italy. No significant differences in incidence of cardiovascular events or case-fatality by social status were found in women when they were analyzed by geographical area.

**Table 4:** Hazard ratio (HR) and 95% confidence intervals (CI) for cardiovascular events by social status indicators classes.

		ALL			NORTH			CENTRE-SOUTH		
		n	p/y	HR^a ^(CI 95%)	n	p/y	HR^a ^(CI 95%)	n	p/y	HR^a ^(CI 95%)
MEN	*Educational level*									
	High	132	25739	Ref.	115	21907	Ref.	17	3832	Ref.
	Medium	184	25303	1.06 (0.83;1.35)	141	18793	0.99 (0.76;1.29)	43	6510	1.23 (0.68;2.19)
	Low	381	39743	1.08 (0.84;1.39)	147	12371	1.41 (1.08;1.82)	234	26372	0.80 (0.47;1.34)
										
	*Marital Status*									
	Married or cohabitating	627	81590	Ref.	348	46915	Ref.	279	34674	Ref.
	Other living arrangements	86	9341	1.16 (0.91;1.49)	58	6444	1.08 (0.80;1.47)	28	2897	1.27 (0.85;1.88)

WOMEN	*Educational level*									
	High	38	32944	Ref.	27	15721	Ref.	11	17223	Ref.
	Medium	75	35647	1.30 (0.84;2.03)	58	23317	0.96 (0.56-1.64)	17	12330	2.05 (0.96-4.37)
	Low	235	67106	1.24 (0.81;1.90)	74	19065	1.23 (0.72-2.11)	161	48041	1.75 (0.89-3.45)
										
	*Marital Status*									
	Married or cohabitating	270	115150	Ref.	130	49214	Ref.	140	65936	Ref.
	Other living arrangements	92	23172	1.18 (0.91;1.52)	35	9495	0.93 (0.62-1.40)	57	13677	1.28 (0.92-1.78)

Men and women 35-69 years old free of previous CVD at baseline (ALL). The population is then stratified by geographical area (NORTH vs. CENTRE-SOUTH of Italy).

n=number of first cardiovascular events. p/y=person-years. Ref.= reference.

^a ^HR for educational levels adjusted for age, systolic blood pressure, total cholesterol, HDL cholesterol, diabetes (yes, no), smoking habits (yes, no), anti hypertensive medication(yes, no). HR for marital status adjusted for age, systolic blood pressure, total cholesterol, HDL cholesterol, diabetes (yes, no), smoking habits (yes, no), anti hypertensive medication (yes, no), education (three levels).

**Table 5:** Hazard ratio (HR) and 95% confidence intervals (CI) for cardiovascular case-fatality by social status indicators classes.

		ALL			NORTH			CENTRE-SOUTH		
		n	p/y	HR^a ^(CI 95%)	n	p/y	HR^a ^(CI 95%)	n	p/y	HR^a ^(CI 95%)
MEN	*Educational level*									
	High	23	1473	Ref.	18	1261	Ref.	5	212	Ref.
	Medium	44	2275	1.12 (0.65;1.92)	29	1683	0.90 (0.46;1.75)	15	592	1.70 (0.55;5.23)
	Low	139	4656	1.06 (0.63;1.78)	25	1717	0.78 (0.41;1.49)	114	2939	1.72 (0.62;4.77)
										
	*Marital Status*									
	Married or cohabitating	175	7691	Ref.	54	4078	Ref.	121	3613	Ref.
	Other living arrangements	41	876	3.2 (2.21;4.64)	21	613	3.41 (1.98;5.86)	20	263	2.61 (1.61;4.24)

WOMEN	*Educational level*									
	High	6	443	Ref.	4	335	Ref.	2	107	Ref.
	Medium	14	852	0.77 (0.28;2.10)	9	724	0.53 (0.13;2.09)	5	128	5.75 (0.97;33.98)
	Low	95	3026	1.01 (0.42;2.37)	21	900	1.07 (0.31;3.72)	74	2126	1.44 (0.34;6.04)
										
	*Marital Status*									
	Married or cohabitating	81	3334	Ref.	27	1590	Ref.	54	1745	Ref.
	Other living arrangements	38	1126	1.15 (0.76;1.75)	9	431	1.13 (0.50;2.54)	29	694	1.15 (0.71;1.87)

Men and women 35-69 years old free of previous CVD at baseline (ALL). The population is then stratified by geographical area (NORTH vs. CENTRE-SOUTH of Italy).

n=number of first cardiovascular events. p/y=person-years. Ref.= reference.

^a ^HR for educational levels adjusted for age, systolic blood pressure, total cholesterol, HDL cholesterol, diabetes (yes, no), smoking habits (yes, no), anti hypertensive medication(yes, no). HR for marital status adjusted for age, systolic blood pressure, total cholesterol, HDL cholesterol, diabetes (yes, no), smoking habits (yes, no), anti hypertensive medication (yes, no), education (three levels).

## Discussion

In Italy, in the past, the risk of cardiovascular events was traditionally considered higher in upper socio-economic classes because people with low social conditions followed a poor but healthier diet, characterized by low levels of saturated fat and high levels of monounsaturated fat and fibres [34]. The Seven Countries study, started in the Sixties by Ancel Keys et al., revealed that this nutritional regime, known as the Mediterranean diet, rich in fruits, vegetables, bread and olive oil was highly protective against death from all-cause and coronary heart disease [35]. We have few direct measures about dietary habits in our cohorts, but, according to periodical national surveys, the Mediterranean diet is diffuse in all socio-economic classes, traditionally in the South and in the Centre of the country, but, nowadays also in the North. According with the same surveys, even the prevalence of the other major risk factors (lipids, smoking and blood pressure) has demonstrated a tendency to become homogeneous during the last half century [36,37]. Data which has not yet been published referred to the 35-74 year-old population of area Brianza (Northern Italy) report an attack rate of 349.07 (x 100,000) for coronary events and 182.77 for strokes in men, 60.70 and 51.38 in women, and a mortality rate of 87.97 for coronary events and 22.96 for strokes in males and 21.95 and 18.23 in females. In Central and Southern Italy the numbers are similar i.e. a little lower but not significantly [38,39]. However, important socioeconomic differences persist between the north and the south of the country. The north is richer and a little more educated. In this paper, the "net" effect of two fundamental social characteristics - education and marital status - on CVD risk factors, incidence and case fatality is reported and commented.

Main cardiovascular risk factors (such as blood pressure, triglycerides, BMI, diabetes) resulted higher in less educated groups in both genders, according to the results of studies conducted in other Western countries [40]. Smoking was more prevalent in men with low educational level, while the opposite tendency was detected in women. This is in agreement with other studies which revealed higher prevalence of tobacco smoking in highly educated women in Southern European countries, while the opposite was true in the Northern Europe [41]. The reason of such difference in smoking between Northern and Southern Europe is likely cultural, being an expression of the process through which the genders progress - or do not progress - to equality. No significant differences in the incidence of cardiovascular events and case-fatality by educational level were found in either gender, when all the cohorts were considered together. However when the Northern cohorts were analyzed separately, a significantly higher incidence appeared in men with a lower level of education compared to men with more education, making these populations more similar to those of Northern Europe and confirming results reported using occupation as the proxy of socioeconomic status [42]. In the Centre and the South of Italy different cardiovascular event risk by educational level was not revealed, but, as mentioned before, in those areas the level of education of the general population was reportedly lower (70% of the men resulted in the low educational level), contributing to the homogenisation of the risk. The incidence differences by education in the North did not produce differences in the rates of cardiovascular incidence and case fatality between the North and the South. This may indicate that while, the influence of education is small, the positive influences may be expected by improving such a variable.

Results confirm the presence of gender differences in the relationship between marital status and cardiovascular risk factors: being married or cohabitating was associated with greater protection in men compared to women [33,43]. As regards the association with cardiovascular events and case-fatality, a threefold higher case-fatality of events was found in men living alone, but not in women. In the Mediterranean culture, the percentage of married or cohabitating men is higher than the percentage in women, and, due to the elevated consideration of family ties, remaining single and living alone (8% of men and 6% of women) is usually not a choice, but probably indicates isolation and the lack of social support. This is particularly true for aging men, more affected by cardiovascular disease occurrence, and less capable of being independent and coping with the stress of life and disease. However, loneliness did not appear to negatively affect women's cardiovascular health and actually, with the exception of smoking, the CVD risk factors resulted lower. Our Mediterranean women seem to better tolerate loneliness, mostly due to widowhood, which results in living alone but does not abolish family ties with children and relatives. These findings in women seem peculiar of our population as they are not reported in studies of Northern populations [44]. It must be noted that the same observation had also been recently reported in a Dutch study, the authors of which attributed their findings to less decisive effects of family bonds in women [45]. If this is a gender issue, culturally or biologically determined, it deserves further exploration. Study limitations include the lack of information in relation to income and occupation that would have been helpful in improving the definition of socioeconomic status. Even though the use of education is simple and characterized by a low non-response rate, it may introduce some degree of bias due to large birth cohort differences in the level of education. In addition, while education is stable over time in an adulthood population, marital status is not. Finally, cardiovascular risk factors were assessed at one point in time.

The major strengths of this study are the inclusion of several cohorts, representative of different socio-cultural contexts in Italy, and the large number of observed events during an average follow-up of 11 years. Moreover, most of the risk factors were not self-reported but directly measured by standardised protocols.

## Conclusions

The analysis here reported evidences that there are similarities, but also important differences, between Northern and Southern Europe in the effects of social factors on cardiovascular risk, event occurrence and mortality. Higher education in Italy has the same protective influences observed in Northern Europe as regards cardiovascular risk factors, while it may be less important for the prevention of disease. It is worth noting the exception of northern Italian men, whose social context is more similar to the northern European one. Family is still a stable institution in Italy and it appears to determine a significant protective effect on men's cardiovascular risk score and a dramatic one on case fatality. In women the effect of cohabitation appears opposed and less pronounced and not at all statistically significant. The lack of cohabitation may not exclude social support and its protective effects. If women, and Mediterranean women in particular, better tolerate loneliness, at least from the point of view of cardiovascular health, is an interesting gender issue which deserves further exploration.

## Competing interests

The authors declare that they have no competing interests.

## Authors' contributions

CF was involved in the statistical analysis of data, made substantial contribution to the interpretation of data and in drafting the manuscript. CD made substantial contribution to statistical analysis and interpretation of data. MAR and GC were involved in the interpretation of data, in drafting the manuscript and revising it critically for important intellectual content.

All other authors contributed to revising the manuscript critically for intellectual content. All authors read and approve the final manuscript.

## Pre-publication history

The pre-publication history for this paper can be accessed here:

http://www.biomedcentral.com/1471-2458/10/574/prepub
